# circRNA_0005529 facilitates growth and metastasis of gastric cancer via regulating miR-527/Sp1 axis

**DOI:** 10.1186/s12860-020-00340-8

**Published:** 2021-01-20

**Authors:** Xing Zhang, Hongwei Yang, Yingdong Jia, Zhengwen Xu, Liuping Zhang, Meng Sun, Jing Fu

**Affiliations:** 1 Department of Gastrointestinal Surgery, Suining Central Hospital, Suining City, 629000 Sichuan Province China; 2 Department of Breast and Thyroid Surgery, Suining Central Hospital, Suining City, 629000 Sichuan Province China; 3 Department of Emergency, Huai’an Hospital Affiliated of Xuzhou Medical University and Huai’an Second People’s Hospital, No. 62 Huaihai South Road, Huai ‘an City, Jiangsu Province China

**Keywords:** Gastric cancer, circ_0005529, miR-527, Sp1, Cell proliferation, Cell migration

## Abstract

**Background:**

Circular RNAs (circRNAs) are endogenous non-coding RNAs, which are associated with various biological processes, including microRNA (miRNA) interaction, protein binding and regulatory splicing. circRNA_0005529 (circ_0005529) is derived from vacuolar protein sorting 33 homologue B (VPS33B), and its biological role in gastric cancer (GC) has not been examined. In this study, the expression and location of circ_0005529 and microRNA-527 (miR-527) were determined by qRT-PCR and fluorescence in situ hybridization (FISH). Cell proliferation and cell migration were determined by MTT, EdU incorporation, colony formation, wound scratch and transwell assays. In addition, immunohistochemistry and western blotting were performed to determine the expressions of specificity protein 1 (Sp1), PCNA, c-myc, E-cadherin and N-cadherin. Western blotting and luciferase reporter assay were performed to study the interaction between circ_0005529 and miR-527 or miR-527 and Sp1. The functional effects of circ_0005529 on GC through regulating Sp1 were further evaluated using xenograft and metastatic mouse models in vivo.

**Results:**

Our results showed that circ_0005529 was upregulated in GC tissues and cells, and had promoting effects on cell proliferation and cell migration. Mechanism analysis suggested that circ_0005529 could bind to microRNA-527 (miR-527) and reduce its expression. The interaction between miR-527 and Sp1 in GC was systematically studied. In addition, the results indicated that Sp1 upregulation could rescue the effects on cell proliferation and migration caused by circ_0005529. Moreover, the inhibitory effects of circ_0005529 downregulation on GC growth and metastasis were evaluated in mouse models. These findings suggested that the axis of circ_0005529/miR-527/Sp1 may serve as a promising treatment target for GC diagnosis and treatment.

**Conclusions:**

These findings suggested that the signal axis of circ_0005529/miR-527/Sp1 may has the potential to be explored as a novel therapeutic target for GC diagnosis and treatment.

**Graphical abstract:**

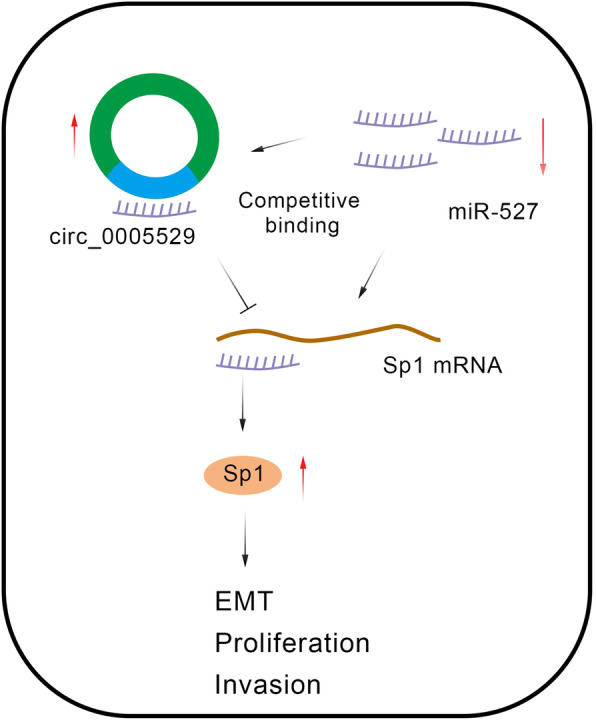

**Mechanism diagram:** During GC development, overexpressed circ_0005529 sponged miR-527 and then upregulated the expression of Sp1. Subsequently, epithelial-mesenchymal transition (EMT), cell proliferation and cell migration were promoted, which ultimately facilitated the tumor metastasis

**Supplementary Information:**

The online version contains supplementary material available at 10.1186/s12860-020-00340-8.

## Background

Gastric cancer (GC) is one of the most common malignancies and the leading cause of cancer-associated death globally [1, 2]. In recent years, major progress has been made in the developments of chemotherapy, surgical treatment and molecular targeted therapy [3, 4]. However, due to the frequent recurrence and metastasis of malignancy, the 5 year prognosis of GC patients is still poor [5]. The complex pathogenesis and biological regulation process bring great challenges to the treatment of gastric cancer [6]. An in-depth understanding of the underlying pathological manner of GC may be beneficial to improve effective targeted therapies.

CircRNAs have a covalent closed loop structure with no 5′ to 3′ polarity, and are related to various biological processes, including microRNA (miRNA) interaction, protein binding and regulative splicing [7, 8]. Through high-throughput sequencing and bioinformatics analysis, more and more circRNAs are identified in GC. circRNA_0005529, derived from alternative splicing of vacuolar protein sorting 33B (VPS33B) transcript, was recently demonstrated to be upregulated in GC tissues based on circRNA chip profiles (GSE78092) [9]. So far, the biological role of circRNA_0005529 in GC remains unclear. Recently, circRNAs were reported to sponge miRNAs to affect the expression of miRNA’s target genes, and further promote cancer development [10]. When exploring novel biomarkers for different pediatric brain tumor subtypes, miR-527 was demonstrated to be significantly overexpressed in low-grade glioma [11]. In addition, miR-527 was identified to suppress lung cancer cell proliferation and migration through targeting TFIIB-related factor 2 (BRF2), an oncogene that is critical for Pol III recruitment and transcription initiation [12]. These findings showed that miR-527 may exert different functions in various tumors. However the effects of miR-527 on GC development are still unclear. As a member of the Sp/KLF family, the transcription factor specificity protein 1 (Sp1) is involved in regulating the expression of genes related to tumor progression-related signal pathways [13, 14]. Sp1 overexpression has been found in many cancers, including thyroid, pancreatic and breast cancers, and its expression was associated with metastatic potential and poor prognosis [15–18]. In GC, Sp1 was strongly expressed, and its activation might serve as a biomarker for poor prognosis and contribute to GC development [19]. Given the apparent overexpression of Sp1, a better insight into the function and underlying mechanism of Sp1 in GC is needed.

In the present study, the expression of circ_0005529 in GC tissues and the effects of circ_0005529 on GC cell proliferation and cell migration were determined. The potential miRNA that may be sponged by circ_0005529 was predicted using Circular RNA Interactome. By using some software for analyzing miRNA’s target genes, miR-527 was predicted to target Sp1 in GC. Therefore, the signal shaft of circ_0005529/miR-527/Sp1 axis in GC was proposed and examined in cells and mouse models, which may provide promising therapeutic target for blocking gastric cancer growth and metastasis.

## Results

### Upregulated circ_0005529 in gastric cancer tissues and cells

**Fig. 1:**
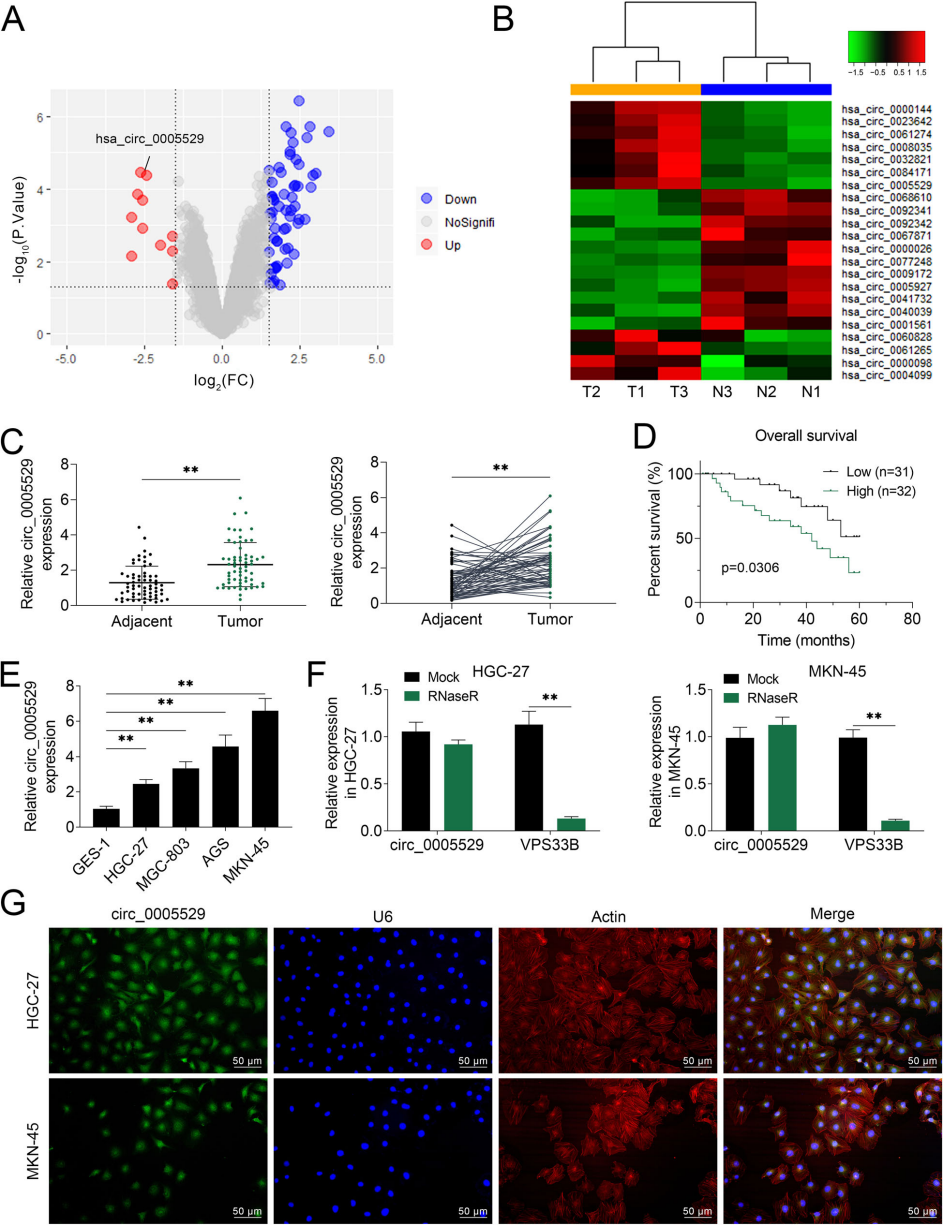
circ_0005529 was up-regulated in gastric carcinoma tissues and cells. **a** Volcano plot of differentially expressed circRNAs. Upregulated and downregulated circular RNAs were screened (red: upregulated, blue: downregulated). **b** Heatmap showing the top 22 significant disordered circular RNAs in GEO: GSE78092; circ_0005529 was upregulated in GC tissues. T-test analysis generated miRNAs that were overexpressed (red, change scale: 0 to 1) and decreased (green, change scale: − 1 to 0) in the GC samples compared to the adjacent samples (0 represents no change). **c** Relative circ_0005529 expression levels in GC tissues (tumor, *n* = 63) and normal adjacent tissues (adjacent, n = 63), as determined using qRT-PCR. **d** Kaplan-Meier curves of overall survival of 63 GC patients, stratified by circ_0005529 expression. **e** Relative circ_0005529 expression levels in human normal gastric mucosal epithelial cells (GES-1) and four gastric cancer cell lines (HGC-27, MGC803, AGS and MKN-45), as determined by qRT-PCR. **f** Relative linear VPS33B mRNA and circ_0005529 expression levels in HGC-27 and MKN-45 cells after RNase R treatment, as determined using qRT-PCR. **g** RNA fluorescence in situ hybridisation for circ_0005529 and Actin was performed in HGC-27 and MKN-45 cells (green, circ_0005529; red, Actin; blue, U6). (Mean ± SEM, ** *p* < 0.01)

To study the role of circRNA in GC, the expression profile containing GC tissues (tumor) and adjacent tissue was analyzed (Fig. 1a). The datasets that generated and analyzed during the current study are available in the [GSE78092] repository, [https://www.ncbi.nlm.nih.gov/geo/query/acc.cgi?acc=GSE78092]. As one of the top differentially expressed circRNAs, circ_0005529 was found to be significantly upregulated in GC tissue compared to adjacent tissue (Fig. 1b). Additionally, a significant upregulation of circ_0005529 in GC tissues was confirmed in another 63 pairs of GC and adjacent tissues (Fig. 1c). GC patients with high circ_0005529 expression had a relatively lower survival rate (Fig. 1d) and a higher probability of poor differentiation, TNM stage and invasion tendency (Table 1). Furthermore, the levels of circ_0005529 in GC cells, such as HGC-27, MGC803, AGS and MKN-45 were higher than that in normal gastric mucosal epithelial cells (GES-1). HGC-27 or MKN-45 cells expressed a relatively lower or higher circ_0005529 expression respectively, and were chosen in the following assays (Fig. 1e). RNase R degrades linear RNA, which has no effect on circular RNA. Accordingly, RNase R degraded the linear transcript of VPS33B, but not circ_0005529 (Fig. 1f). Subcellular localization analysis in Fig. 1g showed that circ_0005529 was mainly expressed in cytoplasm. Together, circ_0005529 is overexpressed in GC tissues and cells, which might play a vital role in GC development.

**Table 1:** Relationship between circ_0005529 and clinico-pathological parameters

Parameters	Number of patients	circ_0005529 expression		χ^2^	*P* value
		Low (< median)	High (≥ median)		
Number	63	31	32		
Age (years)					
< 65	25	11	14	0.450	0.503
≥ 65	38	20	18		
Gender					
Male	38	18	20	0.129	0.719
Female	25	13	12		
Differentiation					
Well	20	15	5	7.800	0.005*
Mederate+Poor	43	16	27		
T stage					
T0-T1	22	15	7	4.870	0.027*
T2-T3	41	16	25		
N stage					
N0-N1	22	16	6	7.483	0.006*
N2-N3	41	15	26		
M stage					
M0	47	27	20	5.028	0.025*
M1	16	4	12		
UCC stage					
I	15	12	3	7.469	0.006*
II-III	48	19	29		
Nerve invasion					
Yes	33	17	16	0.148	0.701
No	30	14	16		
Vessel invasion					
Yes	32	16	17	0.014	0.904
No	25	15	15		

### circ_0005529 promots cell proliferation in GC

**Fig. 2:**
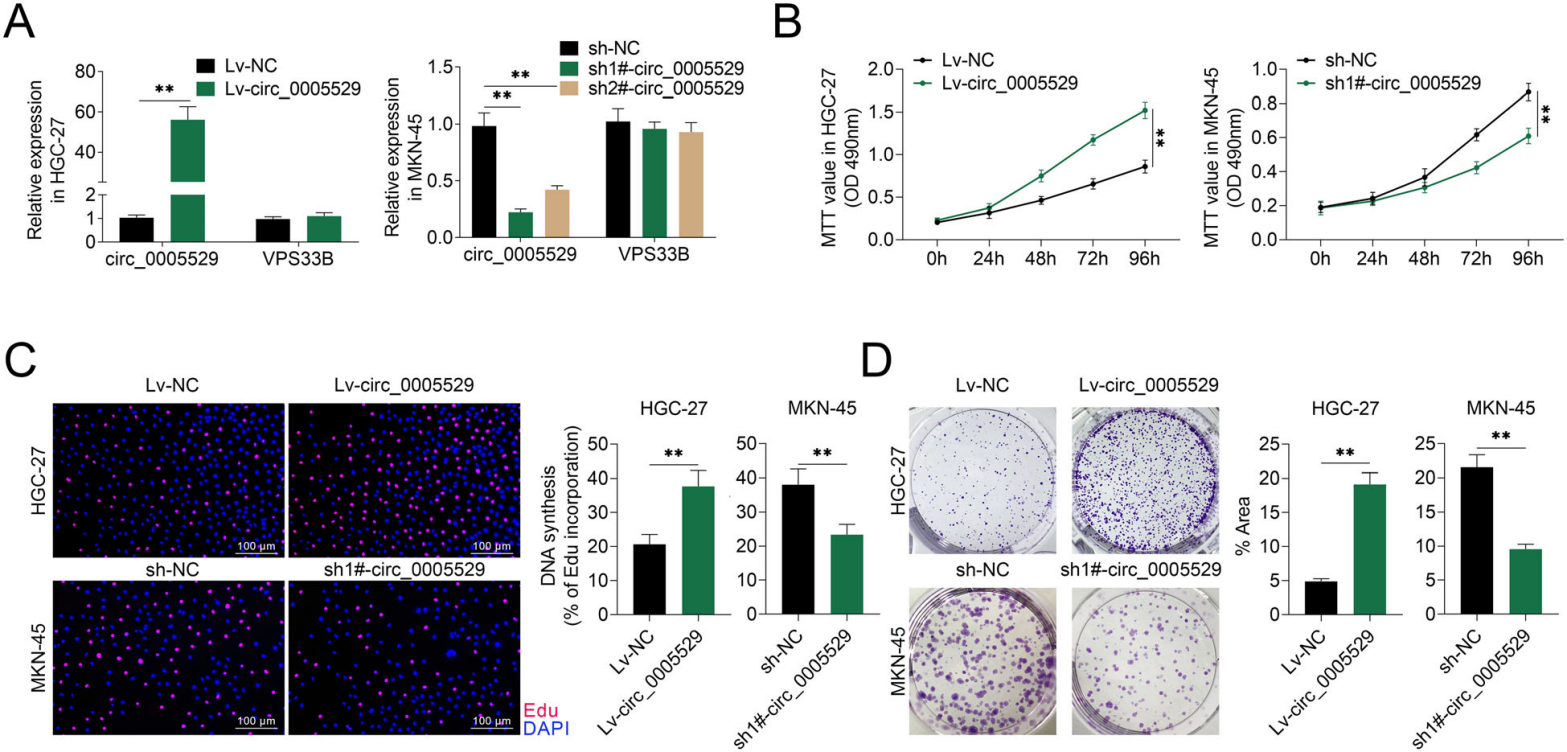
circ_0005529 could promote the proliferation of GC cells. **a** Relative circ_0005529 expression levels in Lv-NC or Lv-circ_0005529 HGC-27 cells and sh-NC or sh-circ_0005529 (sh-#1circ_0005529, sh-#2circ_0005529) MKN-45 cells. **b** Growth curves of HGC-27 cells (Lv-NC or Lv-circ_0005529) and MKN-45 cells (sh-NC or sh-circ_0005529) at 0 h, 24 h, 48 h, 72 h and 96 h. Measurements of the cell growth rate were obtained using a MTT cell proliferation assay kit. **c** Representative profiles of EDU cell growth in HGC-27 cells (Lv-NC or Lv-circ_0005529) and MKN-45 cells (sh-NC or sh-circ_0005529). EDU incorporation rate were quantified. **d** Colony formation analysis of HGC-27 cells (Lv-NC or Lv-circ_0005529) and MKN-45 cells (sh-NC or sh-circ_0005529). Percentage of colony area were quantified. (Mean ± SEM, * *p* < 0.05, ** *p* < 0.01)

To study the function of circ_0005529 in GC, circ_0005529 was overexpressed in HGC-27 cells with relatively lower circ_0005529 expression compared to other GC cell lines, and repressed using shRNAs in MKN-45 cells with a higher expression of circ_0005529. The overexpression and interference effects of circ_0005529 were confirmed by q-PCR (Fig. 2a, Fig. S1A). Subsequently, cell proliferation were examined in HGC-27 cells stably overexpressing circ_0005529 (Lv-circ_0005529), and MKN-45 cells downregulation of circ_0005529 (sh-circ_0005529) as well as their control cells (Lv-NC or sh-NC). The cell growth of HGC-27 cells overexpressing circ_0005529 was promoted compared to the control cells, while downregulation of circ_0005529 suppressed the cell growth of MKN-45 cells (Fig. 2b). In addition, circ_0005529 overexpression in MKN-45 cells, and circ_0005529 inhibition in HGC-27 cells led to an opposite trend for cell curve results (Fig. S1B). The results of EdU analysis indicated that the EdU incorporation rate was upregulated in HGC-27 cells overexpressing circ_0005529. As shown in Fig. 2c, the opposite result was observed in MKN-45 cells downregulation of circ_0005529. Colony formation assay demonstrated that the percentage of colony area was increased in HGC-27 cells overexpressing circ_0005529, whereas decreased in MKN-45 cells down-regulation of circ_0005529 (Fig. 2d). Together, overexpression of circ_0005529 promoted the proliferation of gastric cancer cell line HGC-27 cells, whereas circ_0005529 inhibition suppressed the cell proliferation of MKN-45 cells.

### circ_0005529 promotes cell migration and invasion in GC

**Fig. 3:**
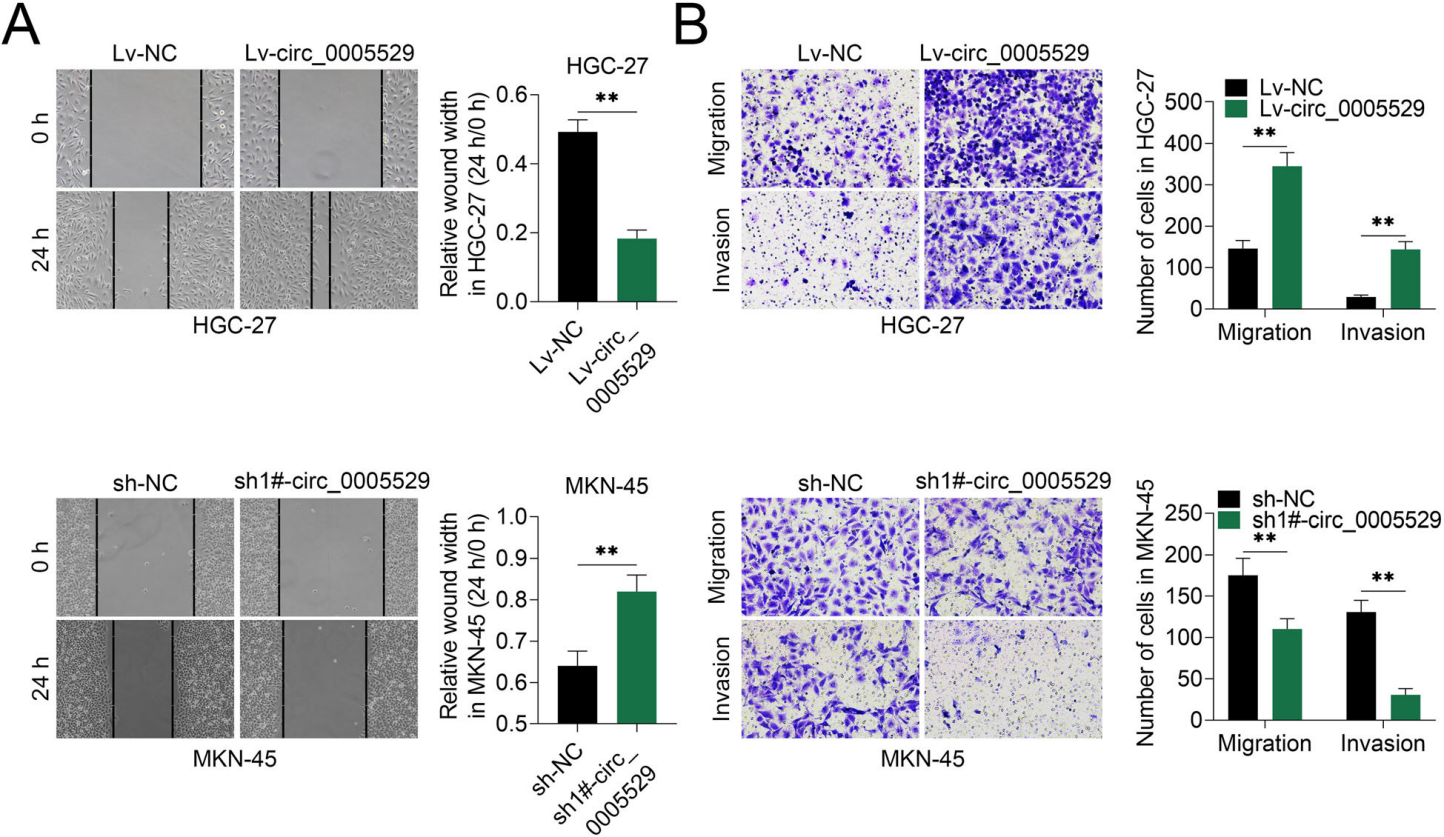
circ_0005529 could promote the migration and invasion of GC cells. **a** Wound scratch healing assay of HGC-27 cells (Lv-NC or Lv-circ_0005529) and MKN-45 cells (sh-NC or sh-circ_0005529). Quantification of the wound-healing assay was shown as histograms. **b** Representative migration and invasion assay images of HGC-27 cells (Lv-NC or Lv-circ_0005529) and MKN-45 cells (sh-NC or sh-circ_0005529). The migrated and invaded cells were quantified and shown as histograms. (Mean ± SEM, * *p* < 0.05, ** *p* < 0.01)

Wound healing and transwell assays were used to study the effects of circ_0005529 on cell migration. As shown in Fig. 3a, overexpression of circ_0005529 promoted the cell migration ability of HGC-27 cells, while down-regulation of circ_0005529 inhibited cell migration of MKN-45 cells. Additionally, the numbers of migrated and invaded HGC-27 cells overexpressing circ_0005529 were significantly increased, whereas markedly decreased in MKN-45 cells down-regulation of circ_0005529 (Fig. 3b). Moreover, transwell results suggested that overexpression of circ_0005529 enhanced the invasion of HGC-27 cells, while downregulation of circ_0005529 inhibited the invasion ability of MKN-45 cells (Fig. S1C). Thus, circ_0005529 overexpression accelerated cell migration in GC, and its suppression exerted inhibitory results.

### Circ_0005529 could sponge miR-527 in GC

**Fig. 4:**
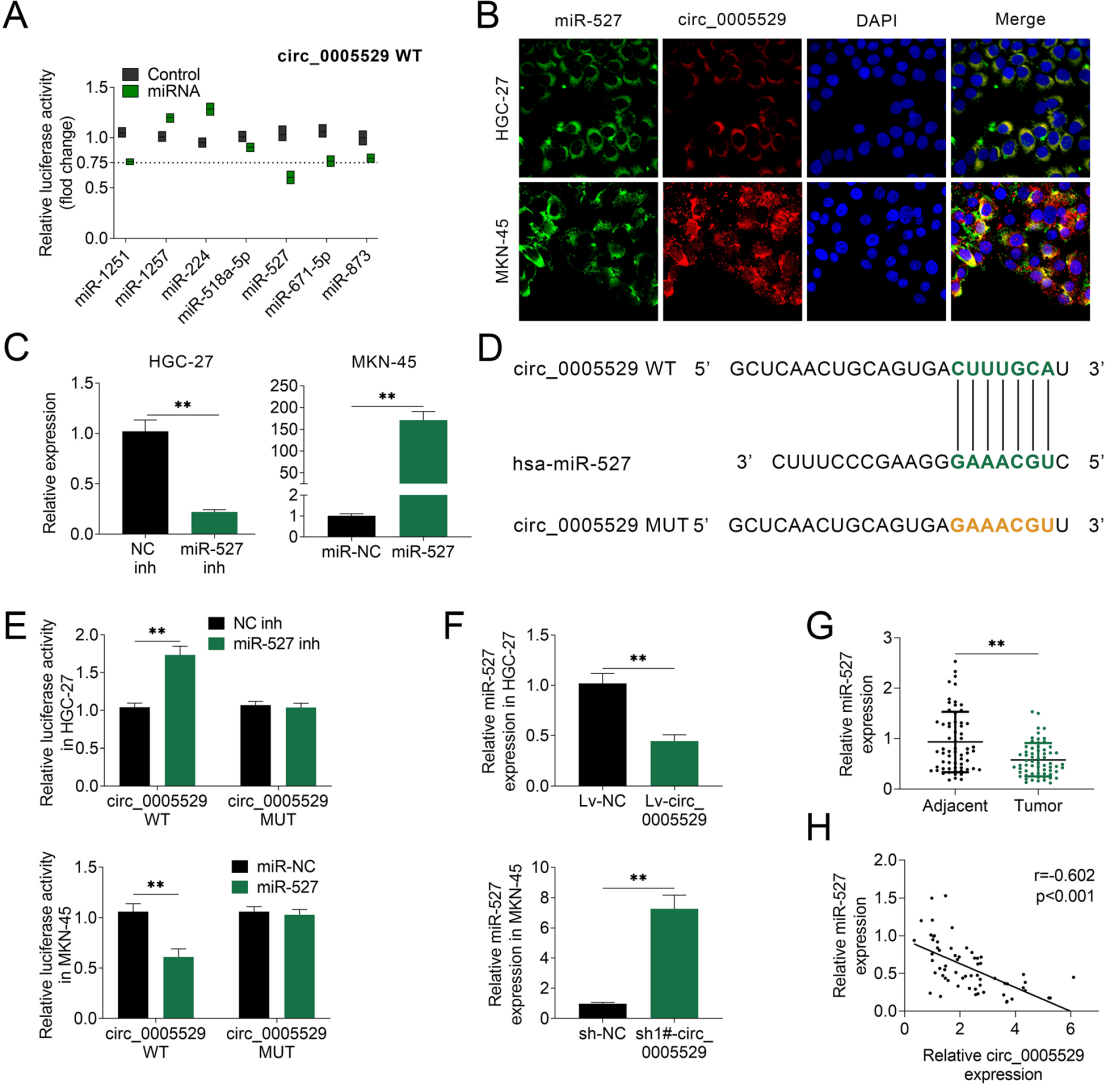
circ_0005529 could sponge and negatively regulate miR-527 expression. **a** Relative luciferase activity of the circ_0005529 reporter plasmid in MKN-45 cells upon transfection of miR-1251, miR-1257, miR-224, miR-518a, miR-527, miR-671-5p and miR-873. **b** RNA fluorescence in situ hybri-disation for circ_0005529 and miR-527 was performed in HGC-27 and MKN-45 cells (green, circ_0005529; red, miR-527; blue, DAPI). **c** Relative miR-527 levels in HGC-27 cells transfected with NC inh or miR-527 inh and in MKN-45 cells transfected with NC mimics or miR-527 mimics, as determined using qRT-PCR. **d** Seeds match for miR-527 in the sequence of circ_0005529. The predicted seed-recognition site in the circ_0005529 sequence and the corresponding miR-527 sequence are marked in green. **e** Relative luciferase activity of the circ_0005529 reporter plasmid in HGC-27 cells upon miR-527 inh or NC inh transfection and MKN-45 cells upon miR-527 mimics or NC mimics transfection. The mutant circ_0005529 reporter was used as a negative control. **f** Relative expression levels of miR-527 in HGC-27 cells (Lv-NC or Lv-circ_0005529) and MKN-45 cells (sh-NC or sh-circ_0005529), as determined using qRT-PCR. **g** Relative miR-527 levels in GC tissues (tumor, *n* = 63) and normal adjacent tissues (adjacent, n = 63), as determined using qRT-PCR. (H) Pearson’s correlation analysis of the relative expressions between miR-527 and circ_0005529 in GC patients. (Mean ± SEM, * *p* < 0.05, ** *p* < 0.01)

It was found that circ_0005529 facilitated cell proliferation and cell migration. However, the underlying mechanism is unknown. Using starBase (http://starbase.sysu.edu.cn/starbase2/), some potential miRNAs were predicted to be targeted by circ_0005529, including miR-1251, miR-1257, miR-224, miR-518a, miR-527, miR-671-5p and miR-873. Among these miRNAs, miR-527 caused the most significant inhibition (about 35%) of the luciferase activity of reporter plasmid containing circ_0005529 sequence (Fig. 4a). The results of FISH also showed that both circ_0005529 and miR-527 were mainly expressed in the cytoplasm of HGC-27 and MKN-45 cells, suggesting the sponge effect between them (Fig. 4b). Additionally, both circ_0005529 and miR-527 were prominently enriched in the immunoprecipitated RNA fractions by the antibody for Ago2 (a miRNA-mediated RISC protein complex, Fig. S1D). As shown in Fig. 4c, miR-527 inhibitor led to a reduced miR-527 expression in HGC-27 cells, and miR-527 mimics increased its expression in MKN-45 cells. Potential base pairing between miR-527 and circ_0005529 was showed in Fig. 4d. As shown in Fig. 4e, the luciferase activity of the circ_0005529 reporter plasmids was increased by miR-527 inhibitor in HGC-27 cells, and reduced by miR-527 in MKN-45 cells. However, mutation of the binding sites eliminates the changes of luciferase activities in these cells. As shown in Fig. 4f, miR-527 was significantly downregulated in HGC-27 cells overexpressing circ_0005529, while markedly upregulated in MKN-45 cells down-regulating of circ_0005529. Furthermore, miR-527 expression was found to be markedly decreased in clinical GC tissues (Fig. 4g). By means of the Pearson correlation analysis, the expression of miR-527 was negatively correlated with circ_0005529 levels in clinical GC tissues (Fig. 4h). In contrast to circ_0005529, the levels of HGC-27, MGC803, AGS and MKN-45 in GC cells were lower than that in GES-1 cells. Compared with other GC cell lines, HGC-27 cells expressed relatively higher miR-527 level, and MKN-45 cells had a lower expression of miR-527 (Fig. S3). The expression of miR-527 was also negatively correlated with the expression of circ_0005529 in HGC-27 and MKN-45 cells. Therefore, circ_0005529 can sponge and downregulate miR-527 in GC.

### miR-527 binds to the 3′-UTR of Sp1

**Fig. 5:**
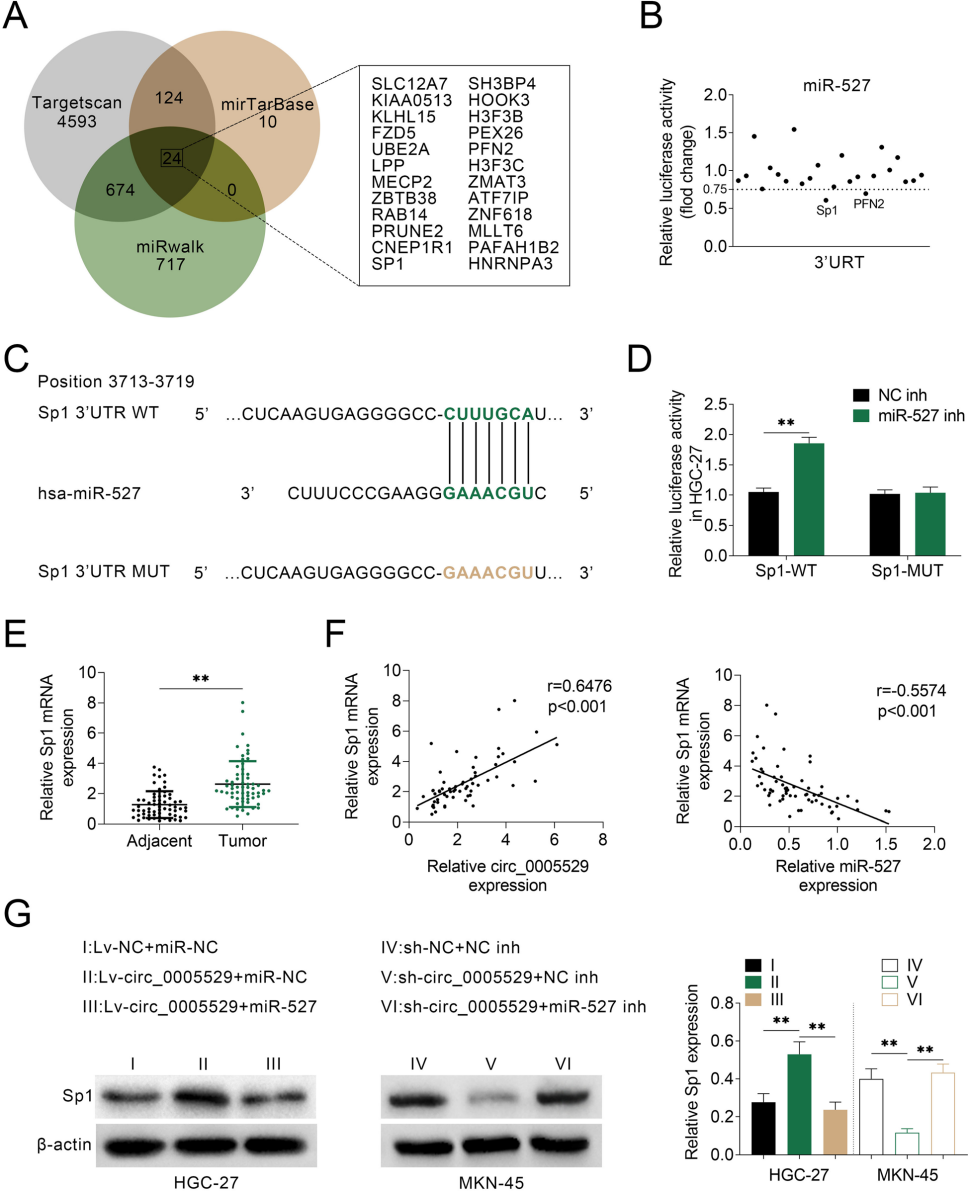
circ_0005529 negatively regulates miR-527 through upregulating Sp1. **a** The intersection of potential genes that could be targeted by miR-527, as predicted by Targetscan, mirTarBase and miRwalk. **b** Relative luciferase activities of the 3′-UTR reporter plasmids (SLC12A7, KIAA0513, KLHL15, FZD5, UBE2A, LPP, MECP2, ZBTB38, RAB14, PRUNE2, CNEP1R1, Sp1, SH3BP4, HOOK3, H3F3B, PEX26, PFN2, H3F3C, ZMAT3, ATF7IP, ZNF618, MLLT6, PAFAH1B2 and HNRNPA3) were measured in MKN-45 cells upon transfection of miR-527 mimics or NC mimics. **c** Seeds match for miR-527 in the 3′-UTR of Sp1. The predicted seed-recognition sites in the Sp1 mRNA sequence and the corresponding miR-527 sequence are marked in green. **d** Relative luciferase activity of the Sp1 3′-UTR reporter plasmid was measured in HGC-27 cells after expressing NC inh or miR-527 inh. The mutant Sp1 3′-UTR reporter was used as a negative control. **e** Relative Sp1 mRNA expression levels in GC tissues (tumor, n = 63) and normal adjacent tissues (adjacent, n = 63), as determined using qRT-PCR. **f** Pearson’s correlation analysis of the relative expressions between circ_0005529 and Sp1 mRNA, miR-527 and Sp1 mRNA in GC patients. **g** Protein expression levels of Sp1 in HGC-27 cells (Lv-NC+miR-NC, Lv-circ_0005529+miR-NC, Lv-circ_0005529+miR-527) and in MKN-45 cells (sh-NC+NC inh, sh-circ_0005529+NC inh, sh-circ_0005529+miR-527 inh), as determined using western blotting. Bands were quantified and shown in histogram. (Mean ± SEM, * *p* < 0.05, ** *p* < 0.01)

The intersection analysis of Targetscan, mirTarBase and miRwalk revealed 24 potential genes of miR-527, as depicted in Fig. 5a. The luciferase activities of reporter plasmids containing 3′-UTR of these genes were determined upon miR-527 overexpression in MKN-45 cells. Among them, the luciferase activity of Sp1 was most significantly inhibited (Fig. 5b).

Figure 5c showed the binding sites between miR-527 and Sp1 3′-UTR. Accordingly, the luciferase activity of the Sp1 3′-UTR reporter plasmids was increased by miR-527 inhibitor in HGC-27 cells. However, mutation of miR-527’s binding sites eliminated the change of luciferase activities (Fig. 5d). Sp1 mRNA level was found to be upregulated in clinical GC tissues (Fig. 5e). Then, a positive correlation between Sp1 mRNA and circ_0005529, and a negative correlation between Sp1 mRNA and miR-527 in GC tissues were indicated (Fig. 5f). Sp1 protein expression was downregulated after overexpression of miR-527 in HGC-27 cells, while increased when miR-527 was downregulated in MKN-45 cells (Fig. S2). Furthermore, Sp1 protein level was upregulated in Lv-circ_0005529 infected HGC-27 cells, and miR-527 mimics rescued circ_0005529-mediated upregulation of Sp1. Accordingly, in sh-circ_0005529 transfected MKN-45 cells, Sp1 expression was significantly decreased, which was rescued by miR-527 inhibitor (Fig. 5g).

### Circ_0005529’s effects on GC cell proliferation and migration were mediated by Sp1

**Fig. 6:**
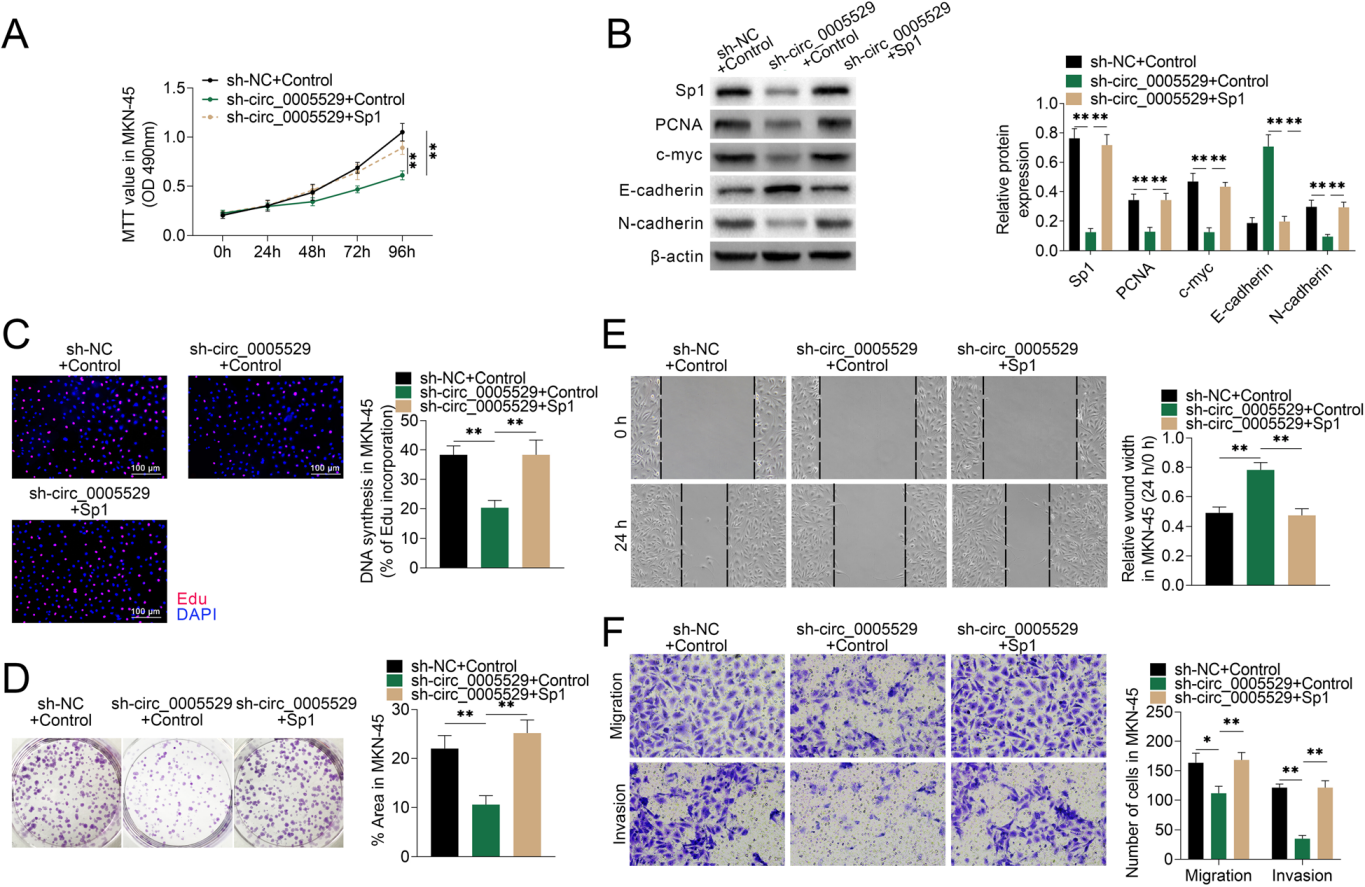
circ_0005529/miR-527 promoted the proliferation and migration of GC cells through regulating Sp1 expression. **a** Growth curves of MKN-45 cells (sh-NC+Control, sh-circ_0005529+Control, sh-circ_0005529+Sp1) at 0 h, 24 h, 48 h, 72 h and 96 h. Measurements of the cell growth rate were obtained using a MTT cell proliferation assay kit. **b** Protein expression levels of Sp1, PCNA, c-myc, E-cadherin and N-cadherin in MKN-45 cells (sh-NC+Control, sh-circ_0005529+Control, sh-circ_0005529+Sp1), as determined using western blotting. Bands were quantified and shown in histogram. **c** Representative profiles of EDU cell growth in MKN-45 cells (sh-NC+Control, sh-circ_0005529+Control, sh-circ_0005529+Sp1). EDU incorporation rate were quantified. **d** Colony formation analysis of MKN-45 cells (sh-NC+Control, sh-circ_0005529+Control, sh-circ_0005529+Sp1). Percentage of colony area were quantified. **e** Wound scratch healing assay of MKN-45 cells (sh-NC+Control, sh-circ_0005529+Control, sh-circ_0005529+Sp1). Quantification of the wound-healing assay was shown as histograms. **f** Representative migration and invasion assay images of MKN-45 cells (sh-NC+Control, sh-circ_0005529+Control, sh-circ_0005529+Sp1). The migrated and invaded cells were quantified and shown as histograms. (Mean ± SEM, * *p* < 0.05, ** *p* < 0.01)

Above results showed that circ_0005529 promoted cell proliferation and migration in GC by sponging miR-527. And miR-527 could target Sp1 in GC. Thus, we next detected whether the effects of circ_0005529 were mediated by Sp1. Overexpression of Sp1 was first confirmed (Fig. S4). Cell proliferation was inhibited in MKN-45 cells down-regulation of circ_0005529, which was restored by upregulation of Sp1 (Fig. 6a). In addition, western blot results showed that protein expressions of Sp1, PCNA, c-myc and N-cadherin were significantly downregulated and E-cadherin was upregulated in MKN-45 cells down-regulating of circ_0005529. However, Sp1 overexpression restored the protein expression changes of these genes (Fig. 6b). Results of EdU incorporation (Fig. 6c) and colony formation (Fig. 6d) assays also indicated that the suppressed cell proliferation could be restored by Sp1 expression. Wound healing analysis indicated that the mobility of MKN-45 cells down-regulating of circ_0005529 was suppressed, which was rescued by Sp1 overexpression (Fig. 6e). Similar results were observed in transwell assays (Fig. 6f). Therefore, circ_0005529 accelerated GC cell proliferation and migration by regulating Sp1 expression.

### Circ_0005529 inhibition suppressed tumor growth and metastasis in vivo

**Fig. 7:**
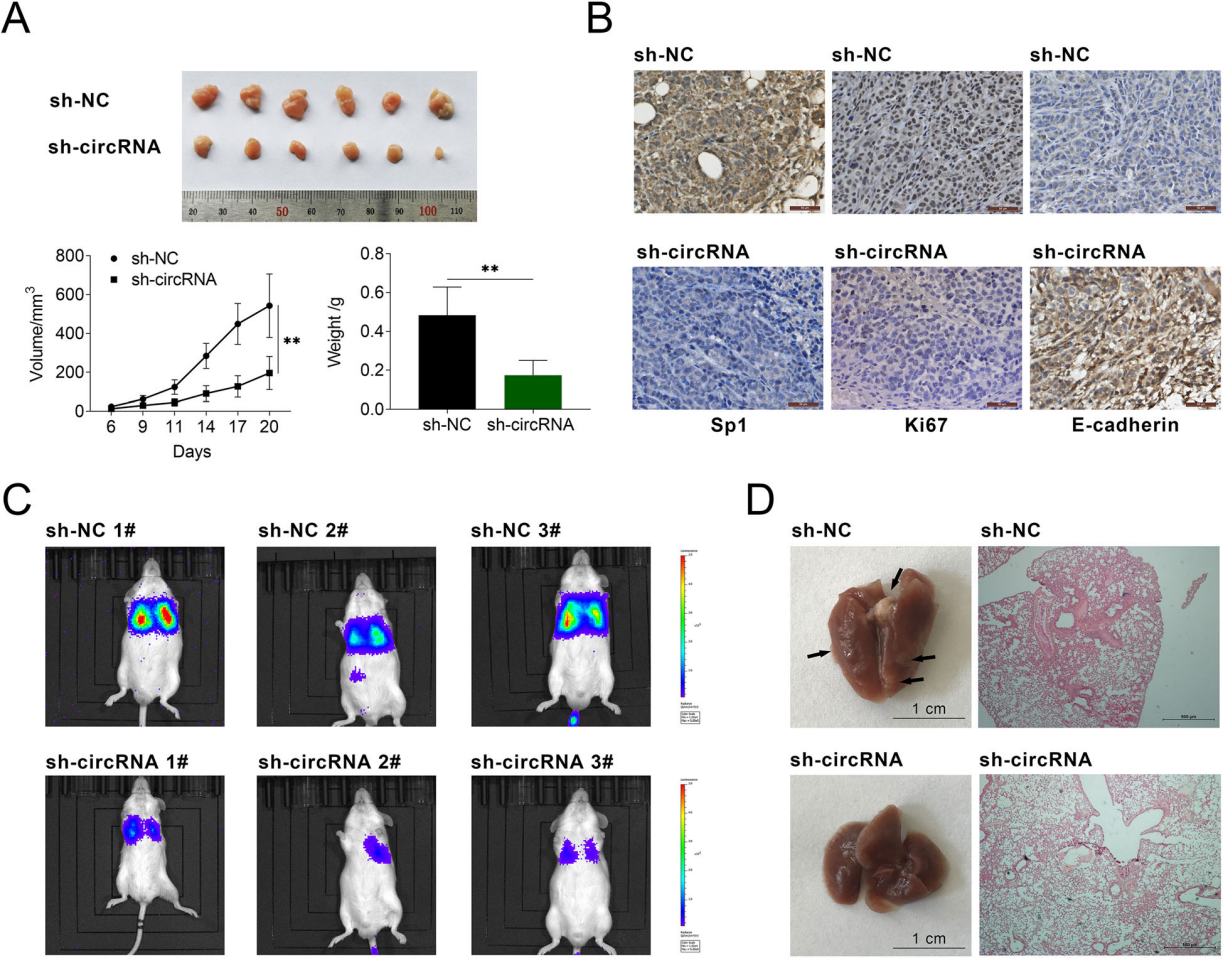
Knockdown of circ_0005529 inhibited GC growth and lung metastasis in vivo. **a** Image of corresponding tumors dissected from mice 21 days post-implantation. Volumes and weight of the xenograft tumors derived from subcutaneous implantation of MKN-45 cells (sh-NC or sh-circRNA). **b** IHC staining of Sp1, Ki67 and E-cadherin in the xenograft tumors derived from subcutaneous implantation of MKN-45 cells (sh-NC or sh-circRNA). Scale bar = 50 μm. **c** Tumor metastasis progression was measured by in vivo luciferase imaging at days 30 after intravenous injection of MKN-45 cells (sh-NC or sh-circRNA). **d** Representative images and H&E staining analysis of lungs dissected from mice with intravenous injection of MKN-45 cells (sh-NC or sh-circRNA). (Mean ± SEM, * *p* < 0.05, ** *p* < 0.01)

Since circ_0005529 inhibition suppressed the cell proliferation and migration in GC cells, the function of circ_0005529 were then explored in mouse models in vivo. To construct the xenograft mouse model, sh-circ_0005529 transfected MKN-45 cells (sh-circRNA) with a luciferase label and the control transfected cells (sh-NC) were subcutaneously implanted into thymic BALB/c nude mice (4–6 weeks, 16-18 g). As shown in Fig. 7a, the corresponding tumors were excised. As shown in the figure, the tumor size of mice in sh-circRNA group was significantly smaller than that in sh-NC group, and the tumor weight of sh-circRNA group mice was also reduced (6/6). The levels of Sp1 and Ki67 levels were significantly downregulated and the expression of E-cadherin level was increased in xenograft tumors in the sh-circRNA group compared to those in sh-NC groups (Fig. 7b). In addition, sh-circRNA induced-tumor inhibition was restored upon Sp1 overexpression (Fig. S1E). To construct the metastatic mouse model, sh-NC and sh-circRNA transfected MKN-45 cells were intravenously injected. By means of an in vivo imaging system and histopathological analysis, sh-circRNA group mice showed a significant lower degree of lung metastasis (Fig. 7c) and a smaller area of tumor nodules in the lung (Fig. 7d), compared to mice of sh-NC group.

## Discussion

In this study, we not only showed the elevated expression of circ_0005529 and its promotional effects on cell proliferation and migration, but also demonstrated a regulatory axis of circ_0005529/miR-527/Sp1. Benefiting from high-throughput sequencing, increasing of circRNAs that involved in GC development were identified. For example, circular RNA cESRP1 acts as a tumor promoter in GC by inhibiting miR-376c-3p to upregulate zinc finger protein 146 (ZNF146) expression [20]. Additionally, circLMTK2 stimulates GC proliferation and metastasis via miR-150-5p/c-Myc pathway [21]. These findings revealed that circRNA-miRNA network plays a crucial role in GC development. As a newly discovered circRNA, circ_0005529 was firstly found to be significantly upregulated in this study. Subsequent analysis predicted and verified that miR-527 could be sponged by circ_0005529 in GC. Since one circRNA might simultaneously bind several miRNAs, whether there are other miRNAs that can be targeted by circ_0005529 reserves further investigation.

As a key biomolecule, miR-527 was also reported to be sponged by other circRNAs. For example, the regulatory network of circ-CDC45/miR-527 has a crucial effect on the proliferation and invasion of glioma cells [22]. BRF2 expression was found to be facilitated by MNX1-AS1/miR-527 axis and contributed to lung cancer development [12]. Although the role of BRF2 has not been characterized in gastric cancer, it can be seen that miR-527 is a biomolecule at a key position in tumorigenesis and development. Besides BRF2, the heparan sulfate 6-O-endosulfatase (SULF2) was also found to be regulated by miR-527. In non-small cell lung cancer (NSCLC), miR-527/SULF2 was reported to suppress TGF-β/SMAD signaling pathway and inhibit the epithelial-mesenchymal transition of NSCLC cells [23]. Due to promoter hypomethylation, SULF2 was identified to be highly expressed in GC tissues and was related to cisplatin sensitivity [24, 25]. In this study, circ_0005529 could affect GC cell proliferation and migration by inhibiting miRNA-527 and regulating the expression of Sp1.Whether circ_0005529/miR-527 affects the cisplatin sensitivity of GC by regulating SULF2 requires further study.

Considering as a general transcription factor for many housekeeping genes, which were associated with cell proliferation and migration, such as c-myc and E-cadherin [26, 27], Sp1 was previously suggested to be post-translationally regulated by multiple modifications, including acetylation, ubiquitylation, phosphorylation, O-linked glycosylation, and SUMOylation [28, 29]. In addition to the above regulations, miRNA-mediated expression regulation is also an important post-transcriptional modification. In the presented study, Sp1 is identified as the main target gene of miR-527, whose expression could restore the circ_0005529 inhibition-induced inhibitory effects on cell growth and migration. Moreover, miR-223 was demonstrated to target Sp1 and inhibit epithelial-mesenchymal transition in GC development [30]. Furthermore, miR-375 and miR-130a-3p have been reported to target Sp1 in glioma to suppress tumor progression and temozolomide (TMZ) resistance [31, 32]. Therefore, it is possible that these four miRNAs regulate the expression of Sp1 in a synergistic way. Considering that Sp1 dominates the expression of many important genes during tumor development, targeting Sp1 may offer novel therapeutic tools for cancer treatment. Natural active ingredients, terameprocol and quercetin, were found to enhance the sensitivity of NSCLC cells to radiation therapy and exert inhibitory effect on prostate cancer cells via inhibiting Sp1 [33, 34]. However, inhibitors that specifically target Sp1 and miRNA mediated-Sp1 inhibition in cancer treatment have not been developed. The vital roles of circRNA and miRNAs in tumor-related pathways highlight their potential to serve as novel therapeutic targets. Our results for the first time demonstrated that miR-527 could negatively regulate Sp1 expression and constitute a regulatory axis of circ_0005529/miR-527/Sp1 in GC. In the future, efforts are needed to develop inhibitors that retard Sp1’s functions based on noncoding RNAs, such as circ_0005529 and miR-527.

In summary, circ_0005529 was upregulated in GC clinical tissues and cells. Cell proliferation and migration were accelerated by upregulation of circ_0005529 in HGC-27 cells and inhibited by circ_0005529 downregulation in MKN-45 cells. circ_0005529 mainly inhibited miR-527 and reduced its expression. In addition, Sp1 was demonstrated as a main target gene of miR-527 in GC, which could restore the effects mediated by circ_0005529. Furthermore, the inhibitory effects of circ_0005529 downregulation on GC growth and metastasis were evaluated in mouse xenograft and metastatic models. Together, the circ_0005529/miR-527/Sp1 axis has the potential to be a novel therapeutic target for GC treatment.

## Methods

### Clinical tissue samples

Paired gastric cancer (GC) tissues (tumor) and adjacent tissues (adjacent) samples were gathered from GC patients who underwent surgical resections at Suining Central Hospital. Patients received no chemotherapy or radiotherapy before section. The study was permissioned by the Medical Ethics Committee of Suining Central Hospital. Written informed consent was acquired. Diagnostics was independently performed by three pathologists. Clinical information of patient are shown in Table 1.

### RNA extraction and circRNA analysis

To extract total RNA, TRIzol reagent (Thermo Fisher Scientific, Rockford, IL, USA) was used. Electrophoresis in agarose was used to evaluate the RNA quality. CircRNAs analysis was conducted in BGI. Tech (Beijing, China, accession codes: GSE78092). After RNase R treatment (2 U/μg, 30 min at 37 °C, Epicentre Technologies, Madison, WI, USA), RNA samples were labeled, hybridized, washed and analyzed by circRNA chips (Arraystar Human circRNAs chip, Rockville, MD, USA).

### Quantitative real-time PCR (qRT-PCR)

A PrimeScript RT Reagent Kit (Takara, Shiga, Japan) was used to synthesize cDNA. SYBR Premix Ex Taq (Takara) was used to test the mRNA expression of target genes. The relative miRNA expressions were quantified by a SYBR PrimeScript miRNA RT-PCR Kit (Takara). GAPDH (glyceraldehyde-3-phosphate dehydrogenase) possesses highly conserved sequence and generally constant expression, thus was chosen to normalize circ_0005529 and Sp1 mRNA levels. To normalize miRNA expressions, U6 snRNA was used. Base on the Ct values of genes and miRNAs, the relative RNA levels were calculated by means of the eq. 2^-ΔΔCT^. Table S1 showed the primer sequences.

### Overall survival analysis

Based on qRT-PCR data, the average level of circ_0005529 served as a criterion. When circ_0005529 level was higher than the average level, “high expression level” was classified. If it was lower than the average value, “low expression level” was identified. Using the Kaplan-Meier method, overall survival curves were depicted according to patients’ follow-up data.

### Cell culture

Human gastric cancer cells of HGC-27, MGC-803 and MKN-45 were obtained from National Infrastructure of Cell Line Resource (Beijing, China). Human normal gastric mucosal epithelial cells (GES-1) were obtained from Procell Life Science&Technology (Wuhan, China). RPMI-1640 Medium (Thermo Fisher Scientific) was used for cell culture of HGC-27, AGS, GES-1 and MKN-45. MGC-803 cells were cultured in Dulbecco’s modified Eagle’s medium (DMEM, Thermo Fisher Scientific). And 10% FBS (Thermo Fisher Scientific) was added in cell culture medium for cell culture, and these cells were cultured in a humidified atmosphere with 5% CO_2_ at 37 °C. An extraction Kit (Inventbiotech, Beijing, China) was used to separate the nucleus and cytoplasm.

### Cell infection and cell transfection

Lentivirus carrying circ_0005529 sequence (Lv-circ_0005529), lentivirus expressing shRNA for circ_0005529 (sh-1#-circ_0005529, sh-2#-circ_0005529) and their controls (Lv-NC or sh-NC) were purchased from GenePharma (Shanghai, China). After cell infection in HGC-27 or MKN-45 cells, puromycin (300 μg/mL, Sigma-Aldrich, St. Louis, MO, USA) was used for cell screening, and qRT-PCR was used to examine the effects of circ_0005529’s overexpression or interference. Cell transfection was performed in HGC-27 or MKN-45 cells using miR-527 mimics (miR-527), miR-1251 mimics (miR-1251), miR-1257 mimics (miR-1257), miR-224 mimics (miR-224), miR-518a-5p mimics (miR-518a-5p), miR-671-5p mimics (miR-671-5p), miR-873 mimics (miR-873), miR-527 inhibitors (miR-527 inh), Sp1 expressing plasmid (Sp1) or the controls (miR-NC, NC inh or Control). miRNA mimics mimic living organisms, and inhibitors matches and inhibits miRNA’s expression, which were obtained by chemical synthesis (GenePharma). By enzyme digestion and ligation, Sp1 expressing plasmid was constructed and then confirmed by DNA sequencing (GenePharma). Cells were seeded in a 6-well plate (5 × 10^4^/per well) 12 h prior to transfection; transfection complexes of Lipofectamine 2000 (Thermo Fisher Scientific), and DNA (1000 ng/per well) or miRNA mimics (or inhibitors, 100 nM/per well) were prepared and incubated (room temperature, 20 min). Cell incubation was then performed with transfection complexes in Opti-MEM medium, and assays for cell function and gene expression were conducted 48–72 h later.

### MTT assay

The cell viability of HGC-27 cells (Lv-circ_0005529 or Lv-NC) or MKN-45 cells (sh-1#-circ_0005529 or sh-NC) was evaluated by the MTT Cell Proliferation Assay Kit (Beyotime, Shanghai, China). Cells were seeded in 96-well plate (5 × 10^3^/per well) and were incubated with CCK-8 solution (10 μl, 2 h at 37 °C) at specific time points. The absorbance was detected by a spectrometer reader at 450 nM (Olympus, Tokyo, Japan).

### 5-Ethynyl-2′-deoxyuridine (EdU) incorporation assay

A Cell-Light EdU DNA Cell Proliferation Kit (RiboBio, Guangzhou, China) was used to perform EdU assays. Cells (1 × 10^4^/well of 96-well plates) were seeded and incubated with EdU (100 μL, 2 h). Then, the cells were cleaned with PBS and stained with Apollo (100 μL, 30 min). After fixed in 4% paraformaldehyde, the cells were stained with DAPI (100 μL, 10 min). After captured images by a fluorescent microscope (Olympus), EdU-positive cells was quantified.

### Colony formation assay

HGC-27 cells (Lv-circ_0005529 or Lv-NC) or MKN-45 cells (sh-1#-circ_0005529 or sh-NC) cells were plated (1 × 10^3^ cells/well of 6-well plates) and cultured at 37 °C for 2 weeks. After that, after fixed, cell colonies were stained by crystal violet (Solarbio, Beijing, China). Colonies were then imaged by an inverted microscope (CK2, Olympus) and quantified using ImageJ.

### Wound healing assay

HGC-27 cells (Lv-circ_0005529 or Lv-NC) or MKN-45 cells (sh-1#-circ_0005529 or sh-NC) cells were grown to 90% confluence. Linear scratch wounds were conducted on the cell monolayer. And the scraped areas were imaged at 0 and 24 h post-scratch by an inverted microscope (Olympus). The distances of the scrape were determined, and the wound healing percentage were analyzed.

### Transwell assay

For cell migration assay, HGC-27 or MKN-45 cells (1 × 10^5^) were cultured in the Transwell upper chamber (BD Biosciences, San Jose, USA). For cell invasion assay, Matrigel (2 mg/ml, BD Biosciences) was used to pre-coat the insert. In the top chamber, cells were cultured with serum-free medium. In the lower chamber, cell culture medium with FBS (10%) was added. Cells in the upper chamber were swabbed off by a cotton swab 24 h later. And the invaded or migrated cells were fixed in methanol, stained by 0.1% crystal violet (Solarbio). Cells were then imaged and counted.

### Luciferase reporter assay

Luciferase reporter assay was conducted as previously reported [35]. Firefly luciferase reporter plasmids containing circ_0005529 or gene 3′-UTR, β-galactosidase expression vector (Promega) and miRNA mimics (miR-527, miR-1251, miR-1257, miR-224, miR-518a-5p, miR-671-5p, miR-873), NC mimics, miR-527 inh or NC inh were co-transfected into HGC-27 or MKN-45 cells, when cell confluence reached 70%. After 24 h, the luciferase activity was measured with a Luciferase Reporter Assay System (Promega, Madison, WI, USA).

### Plasmid construction

Amplified from a human genomic DNA, circ_0005529 sequence was inserted into the p-MIR-reporter plasmid (Promega). The binding sites: CUUUGCA was substituted by GAAACGU and used as a mutant. Similarly, the 3′-UTR p-MIR-reporter plasmids of different genes were obtained. Additionally, the binding sites: CUUUGCA of Sp1 luciferase plasmid was substituted by GAAACGU and used as a negative control. Table S2 showed the relevant primer sequences.

### RNA fluorescence in situ hybridization (FISH)

FITC-labelled miR-527 and Cy3-labelled circ_0005529 probes were commercially obtained from GenePharma. A fluorescence in situ hybridisation (FISH) kit (RiboBio) was used to perform FISH. U6 served as a control for RNA in the nucleus, and Actin was used for cytoplasmic RNA. After fixed in paraformaldehyde, HGC-27 or MKN-45 cells were blocked in pre-hybridization buffer andincubated with probes in hybridization buffer. Followed by DAPI (Sigma-Aldrich) staining, LMS 880 confocal microscope (Carl Zeiss, Germany) was used to photograph fluorescence images.

### RNA binding protein immunoprecipitation (RIP) assay

RIP assay was conducted as previously described {Bierhoff, 2018 #47}. Briefly, cell lysates were obtained from HGC-27 or MKN-45 cells and were incubated with antibodies for Ago2 or IgG. Then, the pulled down complexes were proceeded to qRT-PCR analysis to analyze circ_0005529 or miR-527 levels.

### Western blotting

Radio immunoprecipitation assay (RIPA) protein lysis buffer (Sigma-Aldrich) was used to prepare cell lysates. Protein concentrations were determined using a BCA Protein Assay Kit (Thermo Fisher Scientific). Protein samples (50 μg) were separated by SDS-PAGE and then transferred to PVDF membranes (Millipore, Bedford, MA, USA). The membranes were blocked with skim milk, incubated with primary antibodies (overnight at 4 °C) and a horseradish peroxidase-conjugated secondary antibody (1 h at room temperature). To visualize the immunoreactive bands, the ECL Kit (GE Healthcare, Piscataway, NJ, USA) was used according to the instructions. β-actin served as the loading control. Quantity One software (Bio-Rad, Berkeley, CA, USA) was used to quantify protein bands. Antibody information was listed in Table S3.

### Immunohistochemistry (IHC) assay

After formalin fixation, isolated tumor tissues were embedded by paraffin, and sliced into 5-mm thickness. After immersed in citrate repair solution, the sections were incubated with primary antibody overnight at 4 °C. After that, the sections were stained with secondary antibody for 30 min at room temperature. Finally, 3, 3′-diaminobenzidine (DAB) solution (Sigma-Aldrich) were used to visualize the sections. The IHC images were captured using a microscope (Olympus) and analyzed using ImageJ software. For antibody information, see Table S3.

### Mouse model

Animal experiment methods were approved by the Ethics Committee of Suining Central Hospital. which was in accordance with the Guidelines for the Care and Use of Laboratory Animals published by the National Institutes of Health [36]. To establish the GC xenograft model and lung metastasis model, thymic BALB/c nude mice (4–6 weeks, 16-18 g) were obtained commercially from Vital River Labs (Beijing, China), and were housed in specified pathogen free (SPF) conditions with free access to water and food. MKN-45 cells with circ_0005529 knockdown (sh-circRNA) and the control cells (sh-NC) that carrying a luciferase label were used. For xenograft model, mice (6 mice/group) were randomly allocated to experimental groups and intravenously injected with sh-circRNA or sh-NC cells (1 × 10^6^). The mice were monitored every 3 days for tumor volume (volume = length ×width2/2). 20 days after injection, mice were sacrificed, and tumors were dissected and weighed. For the lung metastasis model, mice were intravenously injected with sh-circRNA or sh-NC cells (5 × 10^5^) and were intraperitoneally injected with D-luciferin (100 μl, 10 mg/mL in PBS, Promega). Then, under anesthesia with 2.5% isofluorane, an IVIS Lumina XR III in vivo imaging system (PerkinElmer, Waltham, CA) was used to image the lung metastatic colonies. Carbon dioxide (CO_2_) administration was used to euthanize mice, and lungs were dissected and photographed. IHC analysis of Sp1, Ki67 and E-cadherin in the tumor tissues from xenograft model was respectively conducted. And lung tissues from mice of lung metastasis model were subjected to H&E (haematoxylin and eosin) staining.

### Statistical analysis

Experiments were conducted for three times independently with more than three samples and presented as the mean ± standard error of the mean (SEM). Data obeyed a normal distribution. Samples and animals of experimental groups were randomly allocated. The allocation process was blind with the investigator. The statistical differences between two groups was analyzed by Student’s t-test, and that among multiple groups was used by one-way ANOVA. By means of Pearson correlation analysis, correlations between circ_0005529, miR-527 and Sp1 were respectively determined. *p* < 0.01 was classified as significant. *, *p* < 0.05; **, *p* < 0.01.

## Supplementary Information

**Additional file 1: Figure S1.** (A) Relative circ_0005529 expression levels in HGC-27 cells (sh-NC or sh-circ_0005529) and MKN-45 cells (Lv-NC or Lv-circ_0005529) cells. (B) Growth curves of HGC-27 cells (sh-NC or sh-circ_0005529) and MKN-45 cells (Lv-NC or Lv-circ_0005529) at 0 h, 24 h, 48 h, 72 h and 96 h. Measurements of the cell growth rate were obtained using a MTT cell proliferation assay kit. (C) Representative migration and invasion assay images of HGC-27 cells (sh-NC or sh-circ_0005529) and MKN-45 cells (Lv-NC or Lv-circ_0005529). The migrated and invaded cells were quantified and shown as histograms. (D) HGC-27 or MKN-45 cells were harvested and mixed with Ago2 antibodies to perform RNA binding protein immunoprecipitation (RIP) assay. circ_0005529 or miR-527 enrichments were tested by qRT-PCR and compared to anti-IgG control. (E) Image of corresponding tumors dissected from mice 6 weeks post-implantation. Volumes and weight of the xenograft tumors derived from subcutaneous implantation of MKN-45 cells (sh-NC+Vector, sh-circ_0005529+Vector, sh-circ_0005529+Sp1). (Mean ± SEM, * *p* < 0.05, ** *p* < 0.01).

**Additional file 2: Figure S2.** Protein expression levels of Sp1 in HGC-27 cells (miR-NC, miR-527) and in MKN-45 cells (NC inh, miR-527 inh), as determined using western blotting. Bands were quantified and shown in histogram. (Mean ± SEM, ** *p* < 0.01).

**Additional file 3: Figure S3.** Relative miR-527 expression levels in human normal gastric mucosal epithelial cells (GES-1) and four gastric cancer cell lines (HGC-27, MGC803, AGS and MKN-45), as determined by qRT-PCR. (Mean ± SEM, ** p < 0.01).

**Additional file 4: Figure S4.** Protein expression levels of Sp1 in MKN-45 cells (Control, Vector, Sp1), as determined using western blotting. Bands were quantified and shown in histogram. (Mean ± SEM, ** p < 0.01).

**Additional file 5. Supplementary Tables.**

## Data Availability

The datasets generated and analyzed during the current study are available in the [GSE78092] repository, [https://www.ncbi.nlm.nih.gov/geo/query/acc.cgi?acc=GSE78092].

## Abbreviations

circRNAs: circular RNAs
miRNA: microRNA
circ_0005529: circRNA_0005529
VPS33B: Vacuolar protein sorting 33 homologue B
miR-527: microRNA-527
Sp1: Specificity protein 1
GC: Gastric cancer
VPS33B: Vacuolar protein sorting 33B
GSE78092: circrna chip profiles
BRF2: TFIIB-related factor 2
