# Supplementary figures and images for: circRNA_0005529 facilitates growth and metastasis of gastric cancer via regulating miR-527/Sp1 axis
Source: BMC Mol Cell Biol. 2021 Jan 20;22:6. doi: 10.1186/s12860-020-00340-8 (PMC7816457; doi:10.1186/s12860-020-00340-8)

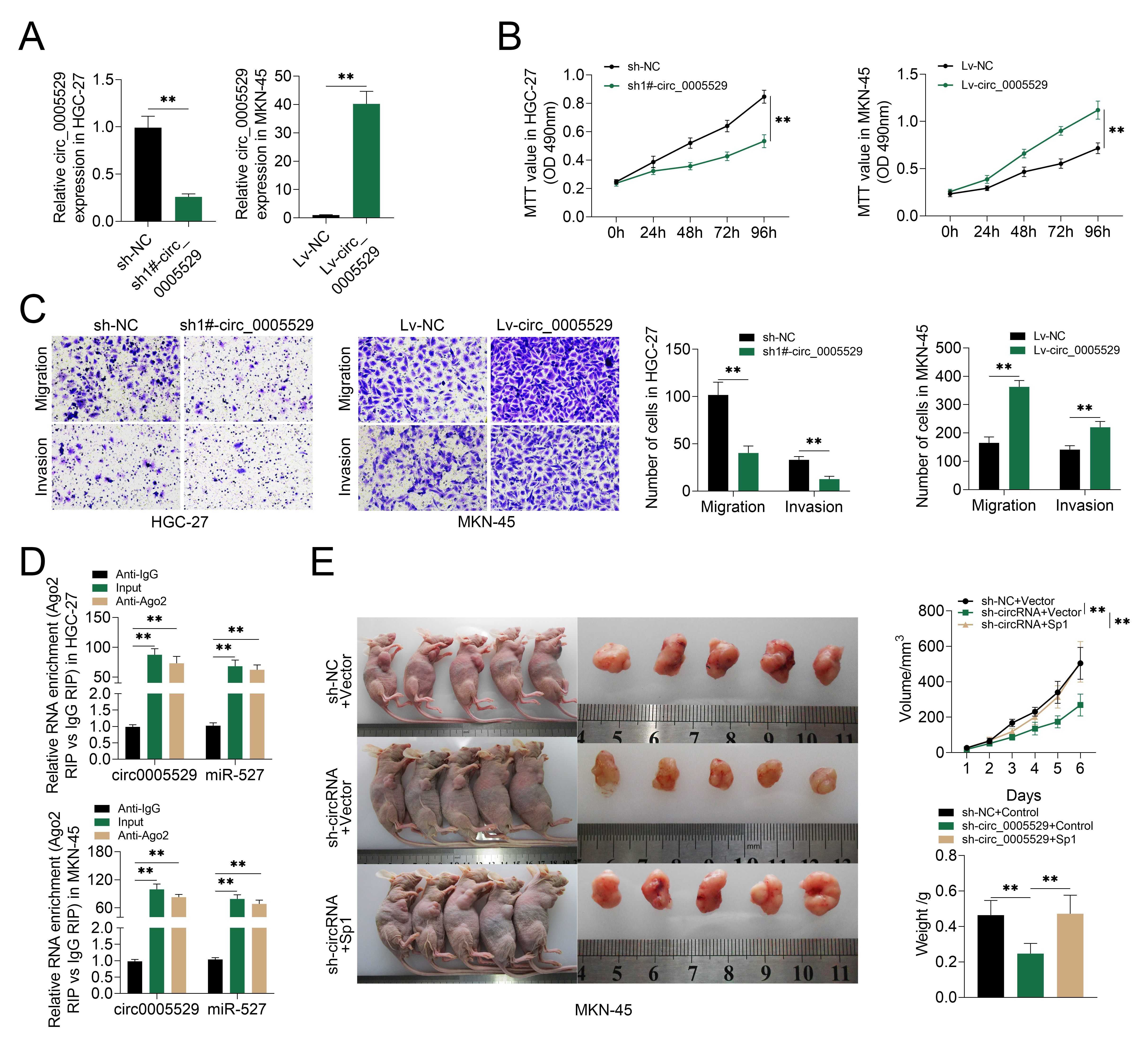

Supplement: Supplementary file 1 — Additional file 1: Figure S1. (A) Relative circ_0005529 expression levels in HGC-27 cells (sh-NC or sh-circ_0005529) and MKN-45 cells (Lv-NC or Lv-circ_0005529) cells. (B) Growth curves of HGC-27 cells (sh-NC or sh-circ_0005529) and MKN-45 cells (Lv-NC or Lv-circ_0005529) at 0 h, 24 h, 48 h, 72 h and 96 h. Measurements of the cell growth rate were obtained using a MTT cell proliferation assay kit. (C) Representative migration and invasion assay images of HGC-27 cells (sh-NC or sh-circ_0005529) and MKN-45 cells (Lv-NC or Lv-circ_0005529). The migrated and invaded cells were quantified and shown as histograms. (D) HGC-27 or MKN-45 cells were harvested and mixed with Ago2 antibodies to perform RNA binding protein immunoprecipitation (RIP) assay. circ_0005529 or miR-527 enrichments were tested by qRT-PCR and compared to anti-IgG control. (E) Image of corresponding tumors dissected from mice 6 weeks post-implantation. Volumes and weight of the xenograft tumors derived from subcutaneous implantation of MKN-45 cells (sh-NC+Vector, sh-circ_0005529+Vector, sh-circ_0005529+Sp1). (Mean ± SEM, * p < 0.05, ** p < 0.01). [file 12860_2020_340_MOESM1_ESM.jpg]

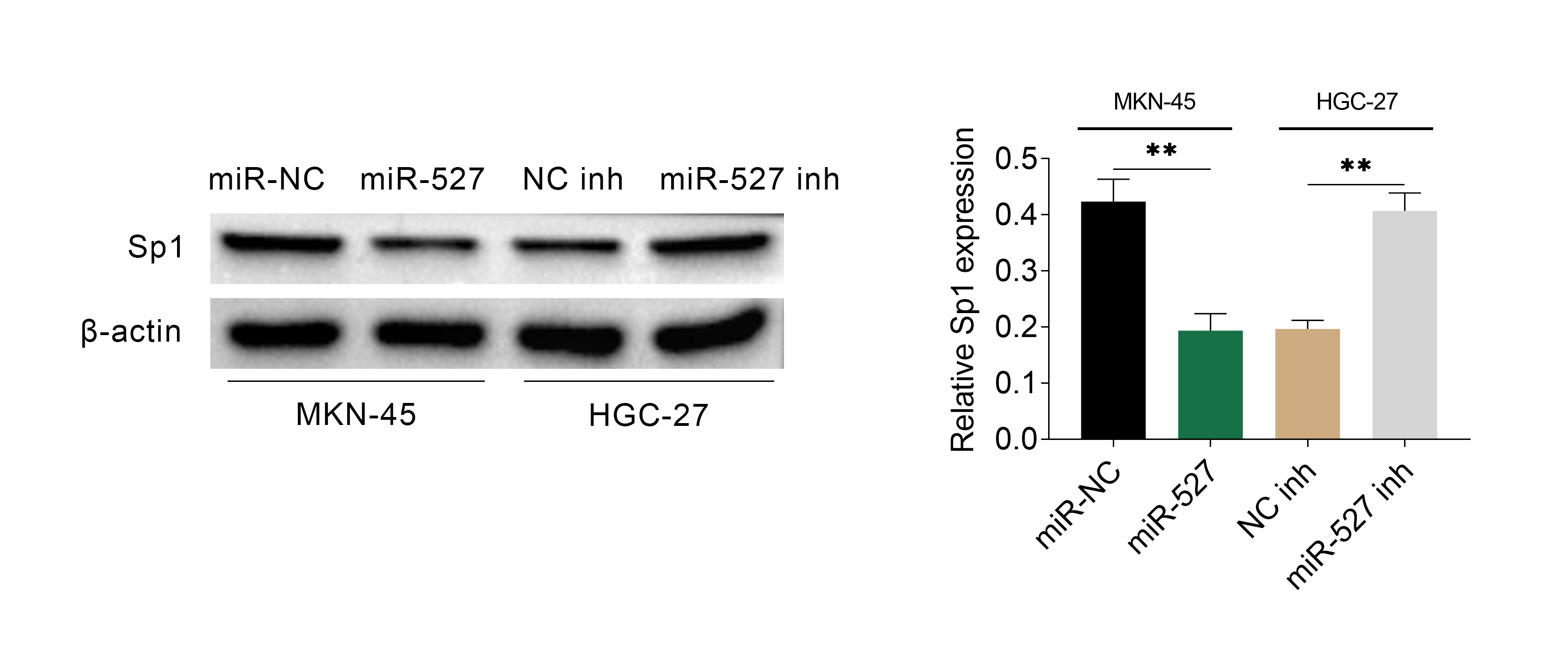

Supplement: Supplementary file 2 — Additional file 2: Figure S2. Protein expression levels of Sp1 in HGC-27 cells (miR-NC, miR-527) and in MKN-45 cells (NC inh, miR-527 inh), as determined using western blotting. Bands were quantified and shown in histogram. (Mean ± SEM, ** p < 0.01). [file 12860_2020_340_MOESM2_ESM.jpg]

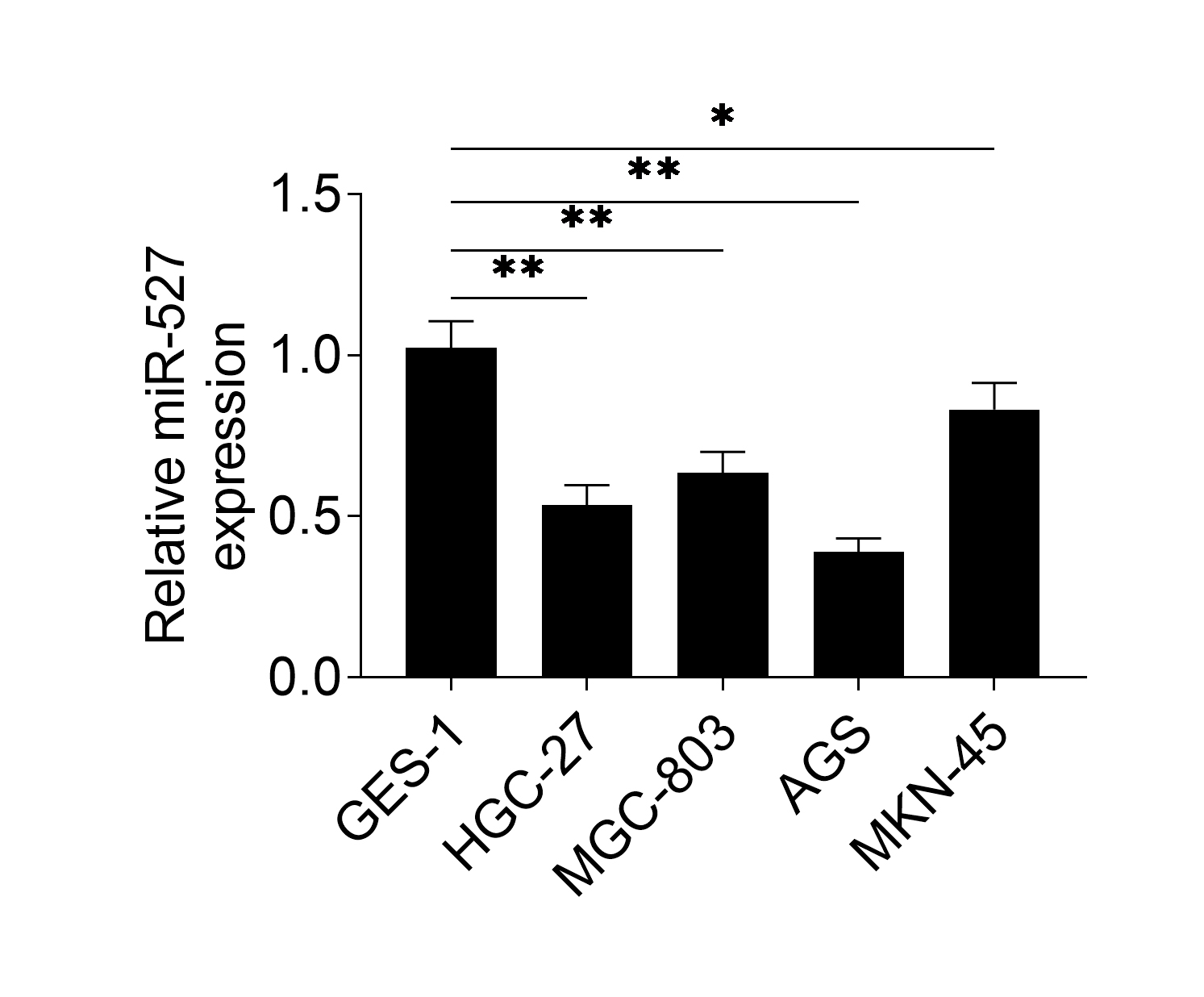

Supplement: Supplementary file 3 — Additional file 3: Figure S3. Relative miR-527 expression levels in human normal gastric mucosal epithelial cells (GES-1) and four gastric cancer cell lines (HGC-27, MGC803, AGS and MKN-45), as determined by qRT-PCR. (Mean ± SEM, ** p < 0.01). [file 12860_2020_340_MOESM3_ESM.jpg]

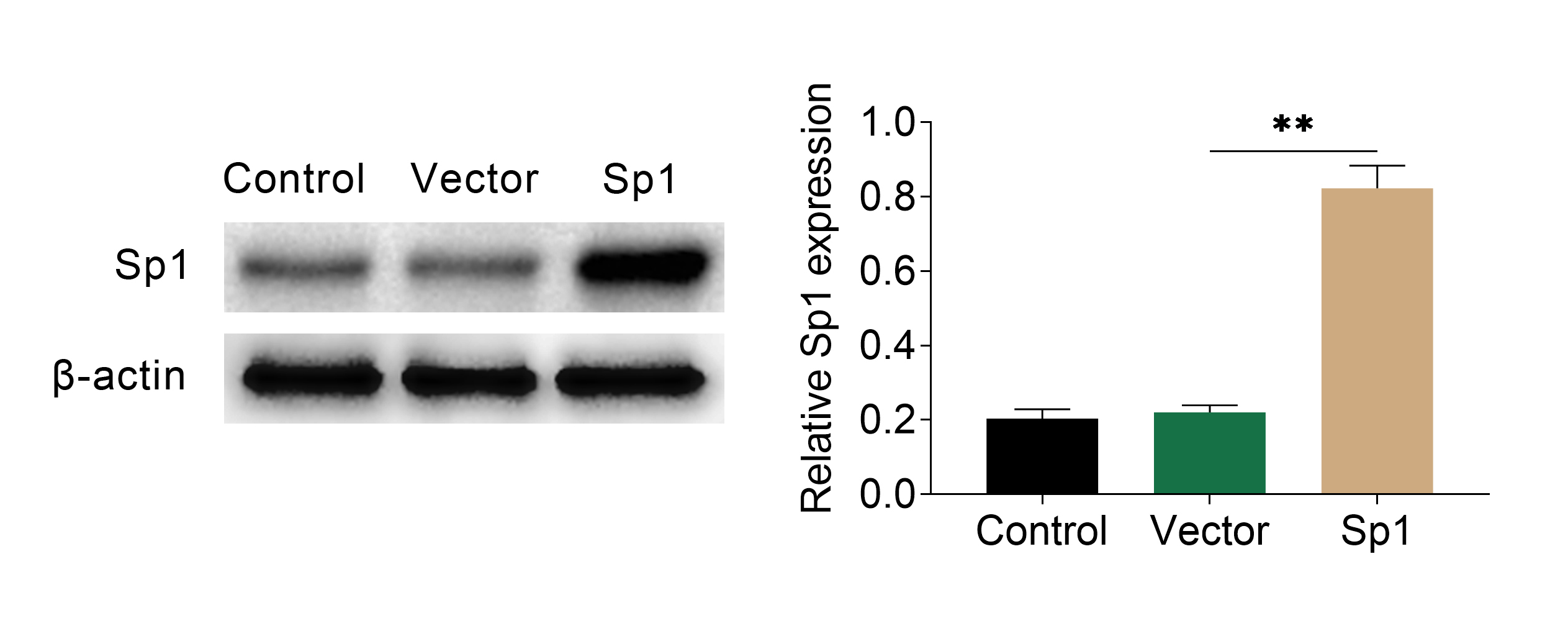

Supplement: Supplementary file 4 — Additional file 4: Figure S4. Protein expression levels of Sp1 in MKN-45 cells (Control, Vector, Sp1), as determined using western blotting. Bands were quantified and shown in histogram. (Mean ± SEM, ** p < 0.01). [file 12860_2020_340_MOESM4_ESM.jpg]
