# Supplementary material for: circRNA_0005529 facilitates growth and metastasis of gastric cancer via regulating miR-527/Sp1 axis
Source: BMC Mol Cell Biol. 2021 Jan 20;22:6. doi: 10.1186/s12860-020-00340-8 (PMC7816457; doi:10.1186/s12860-020-00340-8)
Supplement: Supplementary file 5 — Additional file 5. Supplementary Tables. [file 12860_2020_340_MOESM5_ESM.doc]

**Supplementary Tables**

**Table S1** The primers for qRT-PCR assay.

| **Primer Name** | **Primer Sequence (5’-3’)** |
| --- | --- |
| GAPDH RT primer | random primer |
| GAPDH Reverse primer | AAATGAGCCCCAGCCTTC |
| GAPDH Forward primer | AATCCCATCACCATCTTCCAG |
| circ_0005529 RT primer | random primer |
| circ_0005529 Reverse primer | AGCCGTACCACCTCATCAAG |
| circ_0005529 Forward primer | CTTGGTGGTGTTCTTGGGTG |
| VPS33B RT primer | random primer |
| VPS33B Reverse primer | TTCCTCAAGCACCATCTCAC |
| VPS33B Forward primer | AGACCCCGCATCAAGAATATG |
| Sp1 RT primer | random primer |
| Sp1 Reverse primer | TGTACGTTTGTGCCTCTGTAG |
| Sp1 Forward primer | AGGCTGTGGGAAAGTGTATG |
| U6 RT primer | AACGCTTCACGAATTTGCGT |
| U6 Reverse primer | AACGCTTCACGAATTTGCGT |
| U6 Forward primer | CTCGCTTCGGCAGCACA |
| miR-527 RT primer | CTCAACTGGTGTCGTGGAGTCGGCAATTCAGTTGAGGAAAGGGC |
| miR-527 Reverse primer | TGGTGTCGTGGAGTCG |
| miR-527 Forward primer | ACACTCCAGCTGGGCTGCAAAGGGAAGC |

**Table S2** The primers for plasmid constructs.

| **Primer Name** | **Primer Sequence (5’-3’)** |
| --- | --- |
| circ_102002-p-MIR Reverse primer | GAAGCATGAATTCAAGGTACCCTTTCTCTCTGCCCAGGAACC |
| circ_102002-p-MIR Forward primer | TAATAACTAAGATCTGGTACCGTGCTAGAGCGGCGAAGCT |
| SP1-p-MIR Reverse primer | GAAGCATGAATTCAAGGTACCTTTTTTATTAAAAAACTTTTTAATTGGTTTA |
| SP1-p-MIR Forward primer | TAATAACTAAGATCTGGTACCGATCAGGCACCCGGGGCC |
| SLC12A7-p-MIR Reverse primer | GAAGCATGAATTCAAGGTACCCAACGTACTCAGATTTCATAGGATTTT |
| SLC12A7-p-MIR Forward primer | TAATAACTAAGATCTGGTACCTGCCCAACAGCATCACGG |
| KIAA0513-p-MIR Reverse primer | GAAGCATGAATTCAAGGTACCAAAGTTAGTTTATTTCAGTTTCTACCTTCTG |
| KIAA0513-p-MIR Forward primer | TAATAACTAAGATCTGGTACCGCCCCAGAGGTCGCACTC |
| KLHL15-p-MIR Reverse primer | GAAGCATGAATTCAAGGTACCTCTTCCAACACAAAACCTTCAAAG |
| KLHL15-p-MIR Forward primer | TAATAACTAAGATCTGGTACCTGACATCCTCCTCCCTAAAAAAA |
| FZD5-p-MIR Reverse primer | GAAGCATGAATTCAAGGTACCTTTTTATTGTCTTTACCATTACTTTAATGC |
| FZD5-p-MIR Forward primer | TAATAACTAAGATCTGGTACCGAGGCTGCCGCCGAGGGA |
| UBE2A-p-MIR Reverse primer | GAAGCATGAATTCAAGGTACCTGAAATTTTAAGGTTATTTTTATTTACAACTT |
| UBE2A-p-MIR Forward primer | TAATAACTAAGATCTGGTACCCCCCGGGTACAGTTTAAAGAAG |
| LPP-p-MIR Reverse primer | GAAGCATGAATTCAAGGTACCTAAATGTAATAAGAAGTTTATTGGATTTCTAAA |
| LPP-p-MIR Forward primer | TAATAACTAAGATCTGGTACCATTCAGTCACCTGTTCAGCCGG |
| MECP2-p-MIR Reverse primer | GAAGCATGAATTCAAGGTACCTCACTTTCACAGAGAGATTTATCGAGA |
| MECP2-p-MIR Forward primer | TAATAACTAAGATCTGGTACCCTTTACACGGAGCGGATTGC |
| ZBTB38-p-MIR Reverse primer | GAAGCATGAATTCAAGGTACCTTTTGTTTTATGCTTTTACTTTCCCA |
| ZBTB38-p-MIR Forward primer | TAATAACTAAGATCTGGTACCGTGGCAAGAATTAGAAAAATCTTCAA |
| RAB14-p-MIR Reverse primer | GAAGCATGAATTCAAGGTACCTCCCTCACCACCAAGGTTTTT |
| RAB14-p-MIR Forward primer | TAATAACTAAGATCTGGTACCTGACCTCTTTGCTGTGGCCC |
| PRUNE-p-MIR Reverse primer | GAAGCATGAATTCAAGGTACCAACAGGAAAAAATTATTTAATAGTATAACAAAA |
| PRUNE-p-MIR Forward primer | TAATAACTAAGATCTGGTACCCTGTTGAGAGGCGAGGAGGTAG |
| CNEP1R1-p-MIR Reverse primer | GAAGCATGAATTCAAGGTACCCACGAGTTATATATAAAGTATTTATTTTTAATGC |
| CNEP1R1-p-MIR Forward primer | TAATAACTAAGATCTGGTACCCAATCTTCACTCATTGTTATGGGACT |
| SH3BP4-p-MIR Reverse primer | GAAGCATGAATTCAAGGTACCTTAGGTTTCATGTTTGTTTTATTTAAAGTC |
| SH3BP4-p-MIR Forward primer | TAATAACTAAGATCTGGTACCATGGGTCCCCTCCCCTCC |
| HOOK3-p-MIR Reverse primer | GAAGCATGAATTCAAGGTACCTTTTTATACAAGTCTTTATTTACAACTTGTTTA |
| HOOK3-p-MIR Forward primer | TAATAACTAAGATCTGGTACCAGAAGTTGTGCCGCTCAATCA |
| H3F3B-p-MIR Reverse primer | GAAGCATGAATTCAAGGTACCTTAACTTCAAAAATTTTATTTTAGTCTCATCC |
| H3F3B-p-MIR Forward primer | TAATAACTAAGATCTGGTACCGTGAAGGCAGTTTTTATGGCG |
| PEX26-p-MIR Reverse primer | GAAGCATGAATTCAAGGTACCTGAAGTTAAATTTTTATTATCCTCATTTTACA |
| PEX26-p-MIR Forward primer | TAATAACTAAGATCTGGTACCGGGTCCCTGCGCACCACA |
| PFN2-p-MIR Reverse primer | GAAGCATGAATTCAAGGTACCTTTGGTTGGAATAAATTTATTTCATCT |
| PFN2-p-MIR Forward primer | TAATAACTAAGATCTGGTACCCTGCTAGGCAGACTGTTAAGTATTAGG |
| H3F3C-p-MIR Reverse primer | GAAGCATGAATTCAAGGTACCTTGTGAATGTAAAACATTTAATTTGAAAA |
| H3F3C-p-MIR Forward primer | TAATAACTAAGATCTGGTACCGTGAAGGCAGTTTTTATGGCATT |
| ZMAT3-p-MIR Reverse primer | GAAGCATGAATTCAAGGTACCTTTTCAGACAAGACTCAGAAACCG |
| ZMAT3-p-MIR Forward primer | TAATAACTAAGATCTGGTACCTGATTATCATATTAAGATAGAGCAGCTTTT |
| ATF7IP-p-MIR Reverse primer | GAAGCATGAATTCAAGGTACCGAATGTTAAAAACCCAGTAGTTTTATTTC |
| ATF7IP-p-MIR Forward primer | TAATAACTAAGATCTGGTACCACCTTGGAGCCTTTATATTTTCCTC |
| ZNF618-p-MIR Reverse primer | GAAGCATGAATTCAAGGTACCCCTAAAGGTTGTTTTATTGTAAAACCA |
| ZNF618-p-MIR Forward primer | TAATAACTAAGATCTGGTACCGACTTGACTTCGGGGGAAAAA |
| MLLT6-p-MIR Reverse primer | GAAGCATGAATTCAAGGTACCTTTTTTTTTTTTTTTTTGCAAAACA |
| MLLT6-p-MIR Forward primer | TAATAACTAAGATCTGGTACCATCCACCCTTACCCCTCCTG |
| PAFAH1B2-p-MIR Reverse primer | GAAGCATGAATTCAAGGTACCGAGATGGAGTCTCACTGTCACCCA |
| PAFAH1B2-p-MIR Forward primer | TAATAACTAAGATCTGGTACCGTCACCGCACCAGGCCAT |
| HNRNPA3-p-MIR Reverse primer | GAAGCATGAATTCAAGGTACCAAATTCACAAATGCTAATTTAAATACCC |
| HNRNPA3-p-MIR Forward primer | TAATAACTAAGATCTGGTACCAAACAGCAGAAAAGGGCTACAGTT |

**Table S3** Antibodies used in this study.

| **Antibody name** | **Corporation name** | **Catalog** | **Source** | **Poly/monoclonal** | **Dilution ratio (WB)** | **Dilution ratio**  **(IF/IHC)** | **Concentrations** |
| --- | --- | --- | --- | --- | --- | --- | --- |
| Sp1 | Cell Signaling Technology | 9389 | rabbit | monoclonal | 1:1000 | 1:100 | 200 μg/mL |
| PCNA | Cell Signaling Technology | 13110 | rabbit | monoclonal | 1:1000 | / | 200 μg/mL |
| Ki67 | Cell Signaling Technology | 2586 | mouse | monoclonal | / | 1:100 | 200 μg/mL |
| c-myc | Cell Signaling Technology | 18583 | rabbit | monoclonal | 1:1000 | / | 200 μg/mL |
| E-cadherin | Cell Signaling Technology | 14472 | mouse | monoclonal | 1:1000 | 1:100 | 200 μg/mL |
| N-cadherin | Cell Signaling Technology | 13116 | rabbit | monoclonal | 1:1000 | / | 200 μg/mL |
| β-actin | Thermo Fisher Scientific | 3700 | mouse | monoclonal | 1:1000 | / | 200 μg/mL |
| Goat anti-rabbit IgG | Thermo Fisher Scientific | 31466 | goat | monoclonal | 1:2000 | / | 1 mg/mL |
| Goat anti-mouse IgG | Thermo Fisher Scientific | 31431 | goat | monoclonal | 1:2000 | / | 1 mg/mL |
